# *Myriophyllum aquaticum* Constructed Wetland Effectively Removes Nitrogen in Swine Wastewater

**DOI:** 10.3389/fmicb.2017.01932

**Published:** 2017-10-06

**Authors:** Haishu Sun, Feng Liu, Shengjun Xu, Shanghua Wu, Guoqiang Zhuang, Ye Deng, Jinshui Wu, Xuliang Zhuang

**Affiliations:** ^1^Key Laboratory of Environmental Biotechnology, Research Center for Eco-Environmental Sciences, Chinese Academy of Sciences, Beijing, China; ^2^College of Resources and Environment, University of Chinese Academy of Sciences, Beijing, China; ^3^Key Laboratory of Agro-ecological Processes in Subtropical Regions, Institute of Subtropical Agriculture, Chinese Academy of Sciences, Changsha, China

**Keywords:** constructed wetlands, *Myriophyllum aquaticum*, nitrifiers, denitrifiers, community size, community structure, community interactions

## Abstract

Removal of nitrogen (N) is a critical aspect in the functioning of constructed wetlands (CWs), and the N treatment in CWs depends largely on the presence and activity of macrophytes and microorganisms. However, the effects of plants on microorganisms responsible for N removal are poorly understood. In this study, a three-stage surface flow CW was constructed in a pilot-scale within monospecies stands of *Myriophyllum aquaticum* to treat swine wastewater. Steady-state conditions were achieved throughout the 600-day operating period, and a high (98.3%) average ammonia removal efficiency under a N loading rate of 9 kg ha^-1^ d^-1^ was observed. To determine whether this high efficiency was associated with the performance of active microbes, the abundance, structure, and interactions of microbial community were compared in the unvegetated and vegetated samples. Real-time quantitative polymerase chain reactions showed the abundances of nitrifying genes (archaeal and bacterial *amoA*) and denitrifying genes (*nirS*, *nirK*, and *nosZ*) were increased significantly by *M. aquaticum* in the sediments, and the strongest effects were observed for the archaeal *amoA* (218-fold) and *nirS* genes (4620-fold). High-throughput sequencing of microbial 16S rRNA gene amplicons showed that *M. aquaticum* greatly changed the microbial community, and ammonium oxidizers (*Nitrosospira* and *Nitrososphaera*), nitrite-oxidizing bacteria (*Nitrospira*), and abundant denitrifiers including *Rhodoplanes*, *Bradyrhizobium*, and *Hyphomicrobium*, were enriched significantly in the sediments. The results of a canonical correspondence analysis and Mantle tests indicated that *M. aquaticum* may shift the sediment microbial community by changing the sediment chemical properties. The enriched nitrifiers and denitrifiers were distributed widely in the vegetated sediments, showing positive ecological associations among themselves and other bacteria based on phylogenetic molecular ecological networks.

## Introduction

Water pollution caused by excessive inputs of nutrients from non-point sources is regarded as a serious problem worldwide. According to China’s first pollution survey, the discharge of phosphorus and nitrogen (N) from agriculture accounted for 67 and 57%, respectively, of the total emission loads (Ministry of Environmental Protection, National Bureau of Statistics of China, Ministry of Agriculture, 2010), thereby threatening water quality and human health (Beman et al., 2005). Constructed wetlands (CWs) have been developed as a sustainable technology for diffuse or non-point N pollution of water sources because of their technical feasibility, ecological benefits, and economic advantages (Cronk, 1996; Vymazal, 2011). An important part of the treatment in CWs is attributable to the presence and activity of macrophytes and microorganisms, although until recently the microbial ecology of wetlands has remained relatively uncharacterized. The relative importance of the interactions between the plants and microorganisms responsible for N removal are also poorly understood (Stottmeister et al., 2003).

Wetland macrophytes rooting in anoxic sediments maintain aerobic root respiration via internal oxygen transport through aerenchyma tissue. An excess of oxygen leaking from the roots (known as radial oxygen loss) results in an oxidized rhizosphere (Vartapetian and Jackson, 1997). Oxygen release from the roots of macrophyte species such as *Littorella uniflora*, *Lobelia dortmanna*, and *Glyceria maxima* stimulates nitrification in the rhizosphere compared with that in unvegetated sediment (Bodelier et al., 1996; Petersen and Jensen, 1997; Ottosen et al., 1999). It is generally accepted that plants promote denitrification because they consume oxygen, thereby increasing the anaerobic volume of the soil. Higher carbon availability through root exudation and litter deposition is another important factor that stimulates denitrification (Philippot et al., 2013). Unraveling the plants that affect microbial guilds involved in N cycling has great potential for designing CWs to optimize and compartmentalize the nitrification and denitrification (Ruiz-Rueda et al., 2009).

It is argued that plants alter physicochemical factors, such as the oxygen level, pH, and carbon and N availability, which, in turn, influence the activity, diversity, and abundance of nitrifiers and denitrifiers, although few studies have reported quantitative evidences of these plant–microbe interactions in CWs (Philippot et al., 2013). Recently, several studies, many of which used culture-independent techniques, characterized microbial populations in laboratory-scale units and full-scale CWs under specific conditions (Dong and Reddy, 2010; He et al., 2016; Pelissari et al., 2017). However, the effect of macrophytes on the abundance and structure of microbial communities involved in N removal have not been well studied in CWs (Ruiz-Rueda et al., 2009). In contrast, plants have been shown to induce and stimulate specific microorganisms and create well-defined microbial communities that are involved in nitrification and denitrification in terrestrial soils (Philippot et al., 2002; Patra et al., 2006).

In this study, we designed a three-stage surface-flow CW in pilot-scale to treat high-strength swine wastewater. Macrophytes from a genus known to have the ability to create an oxidized rhizosphere and release organic carbon (*Myriophyllum aquaticum*) (Karjalainen et al., 2001; Laskov et al., 2006) were included. We also demonstrated previously that *M. aquaticum* is able to tolerate high-strength swine wastewater and effectively remove N from polluted waters in laboratory scale tests (Liu et al., 2016). We hypothesized that (i) the macrophytes would shift the structures of microbial communities in both water and sediment and (ii) the microorganisms responsible for N cyclings have been largely enhanced in vegetated condition. To test these hypotheses, high-throughput sequencing of the 16S rRNA gene was conducted to analyze the bacterial communities’ responses to the macrophytes. In addition, real-time quantitative polymerase chain reaction (qPCR) analyses were conducted to determine the effects of the macrophytes on absolute abundance of nitrifying (archaeal and bacterial *amoA*) and denitrifying (*nirS*, *nirK*, *nosZ*) genes. Furthermore, the correlations between bacteria and environmental factors and interactions among bacteria were determined.

## Materials and Methods

### Experimental Setup and Operation Mode

The experiment was conducted at the Jinjing Catchment, Changsha, Hunan Province, China (28°30′ N, 113°18′ E). This site has a subtropical monsoon climate with an annual average rainfall of 1330 mm and an annual average temperature of 17.5°C. A surface-flow CW was established in March 2014, three cells were set up in parallel (three replicates). Each cell was divided into three identical segments (length, 5 m; width, 2 m; and water depth, 0.2 m), named CW1, CW2, and CW3 (Supplementary Figure 1). *M. aquaticum* was planted on the wetland surface at an initial density of 3 kg m^-2^. The cell without planting the *M. aquaticum* was performed as the control. The influents from a storage tank and a fresh water tank were mixed in a settling pond installed in front of the CW, and the effluent of the CW was applied to the land. The characteristics of the swine wastewater are listed in Supplementary Table 1. Each cell was operated in an intermittent flow regime with a total of 0.18 m^3^ d^-1^ wastewater, and the hydraulic retention time was 11 days in each segment.

### Sample Collection

Water samples were collected two or three times per month and were analyzed immediately at the laboratory. The dissolved oxygen (DO) and pH were measured in the field by a multi-parameters water quality monitoring instrument (HACH, HQ30D, United States). Chemical oxygen demand (COD) was measured by a standard potassium dichromate titration method (Federation and Association, 2005). ${NH}_{4}^{+}$-N, ${NO}_{2}^{-}$-N, ${NO}_{3}^{-}$-N, and total N (TN) concentrations were measured by a continuous flow analyzer (AA3; Seal Analytical, Norderstedt, Germany). Microbial samples were sampled at 24 ± 2°C during the stable operation periods. Water (0–5 cm depth) and sediment (0–5 cm depth) samples were taken in the second segment of the CW (CW2, 5–10 m from the inlets), which exhibited the highest N removal efficiency during the monitoring period. In September 2015, sediment cores were taken in 10 replicates from within monospecies of *M. aquaticum* and from unvegetated sediment for molecular analysis. Unvegetated sediment samples were obtained from the upper 5 cm, which included the oxic–anoxic interface. Vegetated sediment samples were obtained from all over the root zone by shaking off sediment that adhered to the roots. All the samples were stored in an ice cooler and brought back to our laboratory. The water samples were filtered onto a 0.22-μm cellulose membrane, and the sediments samples were freeze-dried. Samples for DNA extraction were stored at -80°C until use.

### DNA Extraction and qPCR

Total DNA were extracted using the FastDNA SPIN Kit for Soil (MP Biomedical, Santa Ana, CA, United States) according to the manufacturer’s protocol and then it was visualized using 1% agarose gel electrophoresis and stored at -80°C until use.

Quantification of V4 region of the rrs gene, which encodes the 16S rRNA, archaeal and bacterial *amoA*, *nirK*, *nirS*, and *nosZ*, were performed on a real-time PCR system (Bio-Rad, Hercules, CA, United States) using SYBR green as a fluorescent dye. The thermal cycling conditions and primers used for each reaction are described in Supplementary Table 2. Gene copy numbers in unknown samples were determined based on standard curves obtained from 10-fold serial dilutions of plasmids containing the target genes. The employed results with correlation coefficient and amplification efficiency were greater than 0.98 and 98%, respectively. All samples were run on an agarose gel after the reaction to confirm the size of amplicons, and the specificity of the amplification products was confirmed by a melting curve analysis.

### PCR Amplification, Product Purification, and MiSeq Sequencing

The V4 region of the bacterial 16S rRNA gene was amplified using the primers 515F (5′-GTGCCAGCMGCCGCGGTAA-3′) and 806R (5′-GGACTACHVGGGTWTCTAAT-3′) (Caporaso et al., 2012). Each pair of primers used to amplify a certain water or sediment sample was barcoded with a sample-identifying 12-base barcode on both the forward and reverse primers. The 50 μL reaction system contained 20–30 ng of DNA, 1.5 μL of each primer (at a final concentration of 0.3 μM), and 25 μL of Premix Ex Taq (TaKaRa, Dalian, China). The PCR conditions, performed in a PCR instrument (Eastwin, China), consisted of an initial denaturation step at 94°C for 1 min, followed by 30 cycles at 94°C for 20 s, 57°C for 25 s and 72°C for 30 s, and a final extension step at 72°C for 10 min. The PCR amplicons were purified with the Gel Extraction Kit (D2500-02, Omega Bio-Tek, Norcross, GA, United States), and DNA concentrations were quantified by a spectrophotometer (NanoDrop Technologies, Wilmington, DE, United States). True positive amplicons were combined equally, and a DNA library was obtained according to the MiSeq Reagent Kit Preparation Guide (Illumina, San Diego, CA, United States), and then the DNA library was sequenced using the Illumina MiSeq platform according to the manufacturer’s instructions. All the raw sequences have been deposited into NCBI Short Read Archive (SRA) with accession no. SRP095281.

After assigning each sequence to its sample according to its barcode, allowing up to one mismatch, a total of 675,089 reads from both ends were obtained as a partitioned run for the 40 samples. Paired-end reads of sufficient length (those with at least a 30 bp overlap) were combined into full-length sequences with targeting 253 bp as average fragment length by FLASH (Magoč and Salzberg, 2011). Thereafter, quality trimming was performed using Btrim with a quality score of 20 as cutoff and 5 as window size (Kong, 2011). After removing the sequences with ambiguous bases, the sequences with lengths out of 245–260 bp were removed. Thereafter, UPARSE program (Edgar, 2013) was used to remove the chimeras and classify the sequences into operational taxonomy units (OTUs) at the 97% similarity level. The taxonomic annotation of individual OTUs was performed through Ribosomal Database Project (RDP) classifier (Wang et al., 2007) with a minimal 50% confidence score. Finally, random re-sampling was performed with 10,000 sequences per sample, and this resampled OTU summary table was used for further statistical analysis (Supplementary Data Sheet 2).

### Statistical Analysis

One-way analysis of variance (ANOVA) followed by Student–Newman–Keuls test was used to check for quantitative differences between treatments using Statistics for Windows version 20.0 (IBM Corp., Armonk, NY, United States). The dataset generated by 16S rRNA gene sequencing was further analyzed with all of the following statistical methods. (1) α-diversity comparison analysis was performed using R vegan package. (2) Comparison tests between two groups were based on unpaired Student’s *t*-test in Excel software. (3) The canonical correspondence analysis (CCA) was used to evaluate the linkages compositions and environmental factors (Legendre and Legendre, 2012). To select environmental factors in the CCA modeling, we used variation inflation factors to examine whether the variance of the canonical coefficients was inflated by the presence of correlations with other attributes. If a factor had a variation inflation factor value greater than 20, we deemed it to be affected by other attributes and consequently removed it from the CCA biplot (Yang et al., 2014). Multivariate testing, based on 999 Monte Carlo permutations, confirmed the significance of the two canonical axes (*p* ≤ 0.001). The importance of the environmental attributes was verified by Mantel tests with 999 permutations based on their significant correlation (*p* ≤ 0.001). (4) Phylogenetic molecular ecological networks (pMENs) were constructed by regularly a random matrix theory (RMT)-based interface approach to elucidate the microbial interactions in the sediments and water with and without *M. aquaticum*. pMENs were generated based on a resampled OTU table at Molecular Ecological Network Analysis Pipeline (Zhou et al., 2010, 2011; Deng et al., 2012)^1^. The constructed pMENs were visualized using Cytoscape 3.30 software.

## Results

### Treatment Performance

**FIGURE 1:**
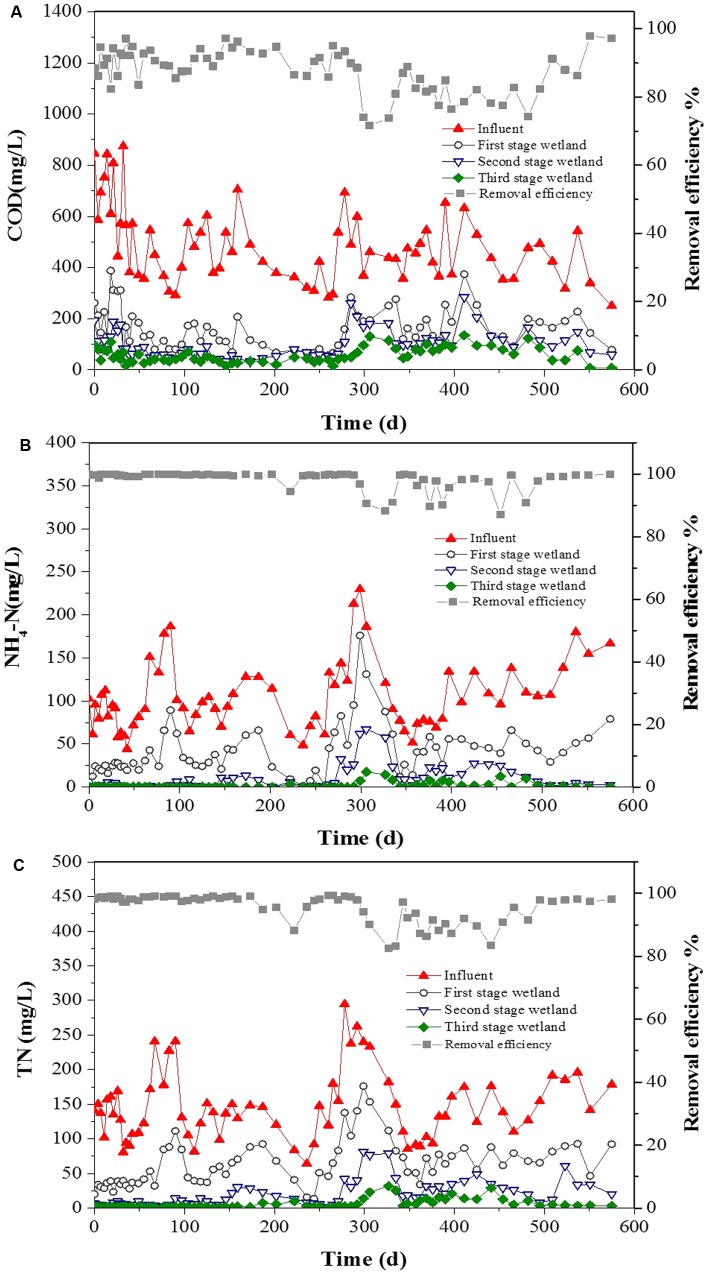
Treatment performances in the three-stage CW. **(A)** COD; **(B)** ammonium; and **(C)** TN.

During the operation (600 days; **Figure 1**), the three-stage CW achieved COD removal efficiency of 88.2% under an average inflow concentration of 484 mg L^-1^. More than 75% of removal occurred in the first-stage wetland, which had the highest removal efficiency of 66.5%. The average inflow ${NH}_{4}^{+}$-N concentration was 107 mg L^-1^, accounting for more than 70% of the N inputs to the wetland. The average ${NH}_{4}^{+}$-N removal efficiency was 98.3%. The average TN concentration of 150 mg L^-1^ in the wastewater inflow was reduced to an average effluent level of 5.9 mg L^-1^, which achieved removal efficiency of 95.8%. The highest removal efficiencies of ${NH}_{4}^{+}$-N and TN (78.6 and 71.6%, respectively) were achieved in the second-stage CW. These performances were stable during the operation periods, even with the high average COD, ${NH}_{4}^{+}$-N, and TN removal efficiencies of 92.3, 99.5, and 97.9%, respectively, in winter.

### Change of Abundances of 16S rRNA and Functional Marker Genes by *M. aquaticum*

**FIGURE 2:**
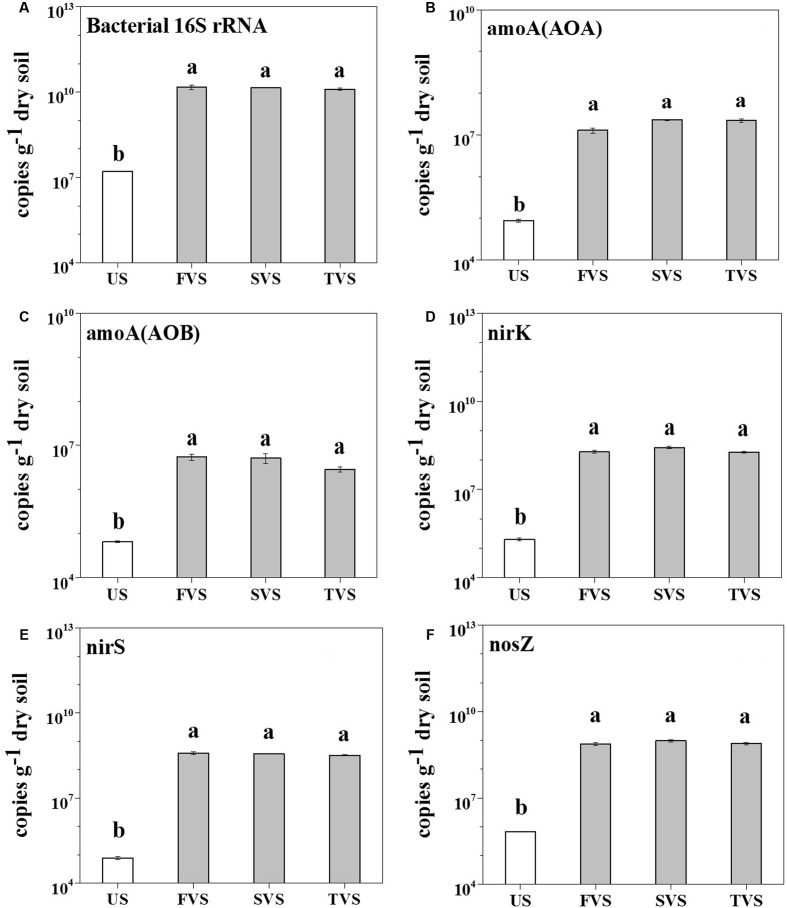
Abundances of the bacterial 16S rRNA **(A)**, archaeal *amoA* **(B)** and bacterial *amoA* **(C)**, *nirK* **(D)**, *nirS* **(E)**, and *nosZ* **(F)** genes. US, sediment without *M. aquaticum*; FVS, sediment with *M. aquaticum* in the first-stage wetland; SVS, sediment with *M. aquaticum* in the second-stage wetland; and TVS, sediment with *M. aquaticum* in the third-stage wetland.

The V4 region of the rrs gene, which encodes for the 16S rRNA, archaeal and bacterial *amoA*, and *nirK*/*nirS*/*nosZ* genes, was quantified by qPCR to demonstrate the abundances of total bacteria, ammonia-oxidizing archaea (AOA), ammonia-oxidizing bacteria (AOB), and denitrifiers. In the sediment, the results showed that the abundance of the bacterial 16S rRNA gene was increased significantly from 1.61 × 10^7^ to 1.44 × 10^10^ copies per gram dry soil (862-fold) by *M. aquaticum* (**Figure 2**). The abundance of archaeal *amoA* gene increased significantly from 8.85 × 10^4^ to 1.93 × 10^7^ copies per gram dry soil (218-fold), and the abundance of the bacterial *amoA* gene increased from 6.65 × 10^4^ to 5.75 × 10^6^ copies per gram dry soil (86-fold). The archaeal *amoA* gene abundance was more increased than the bacterial *amoA* abundance, and it outcompeted the bacterial *amoA* gene in all sediments containing *M. aquaticum*. However, there were no significant differences between the abundances of archaeal and bacterial *amoA* genes in the unvegetated sediment. For denitrifying genes, *M. aquaticum* significantly increased the abundance of *nirK* gene from 2.03 × 10^5^ to 2.16 × 10^8^ copies per gram dry soil (1,063-fold), while the abundance of *nirS* gene increased significantly from 7.73 × 10^4^ to 3.58 × 10^8^ copies per gram dry soil (4,620-fold). In the unvegetated sediment, the *nirK* outcompeted *nirS* gene. However, a greater abundance of the *nirS* gene, compared with that of the *nirK* gene, was detected in sediments containing *M. aquaticum. M. aquaticum* significantly increased the abundance of *nosZ* gene from 6.84 × 10^5^ to 8.31 × 10^8^ copies per gram dry soil (1,210-fold), while in the water (Supplementary Figure 2), *M. aquaticum* significantly decreased the abundance of the bacterial 16S rRNA gene from 1.66 × 10^12^ to 3.79 × 10^10^ copies per liter water (44-fold), and the bacterial *amoA* decreased by one order of magnitude. The abundances of archaeal *amoA* and denitrifying genes in the water showed no significant response to *M. aquaticum* (*p* > 0.05).

### Shift of the Microbial Community Composition by *M. aquaticum*

MiSeq high-throughput sequencing of the bacterial 16S rRNA gene was performed to explore the effect of *M. aquaticum* on the structure of functional communities. In the unvegetated zone, α-diversity indices (Supplementary Table 3) did not differ between sediments and water (*p* > 0.05). The sediments containing *M. aquaticum* showed significantly higher bacterial species richness compared with the unvegetated sediment. The composition of bacterial communities in the sediment with *M. aquaticum* differed significantly from those in the unvegetated sediment based on a principle coordinates analysis (Supplementary Figure 3).

The relative bacterial community abundances were analyzed at the phylum level (Supplementary Figure 4). *Firmicutes* was the most abundant phylum in the unvegetated sediment and water, accounting for 43.1 and 50.6%, respectively, of the total effective bacterial sequences. In the sediment and water containing *M. aquaticum*, *Proteobacteria* was significantly enriched (*p* < 0.001), and it was the most abundant phylum, accounting for 23.73 and 46.65%, respectively, of the total effective bacterial sequences. This result is similar to the previous findings of bacterial communities in constructed and natural wetlands, in which *Proteobacteria* was also the most dominant community (Wu et al., 2016). Within the *Proteobacteria*, *Alpha-*, *Beta-*, and *Gamma-Proteobacteria* were all enriched substantially in sediment and water containing *M. aquaticum* (Supplementary Figure 5). The other dominant phyla in the sediment containing *M. aquaticum* were *Firmicutes* (13.1%), *Acidobacteria* (11.9%), *Planctomycetes* (11.0%), *Chloroflexi* (11.5%), *Actinobacteria* (9.1%), *Euryarchaeota* (3.1%), *Bacteroidetes* (1.9%), and *Nitrospirae* (0.6%).

**FIGURE 3:**
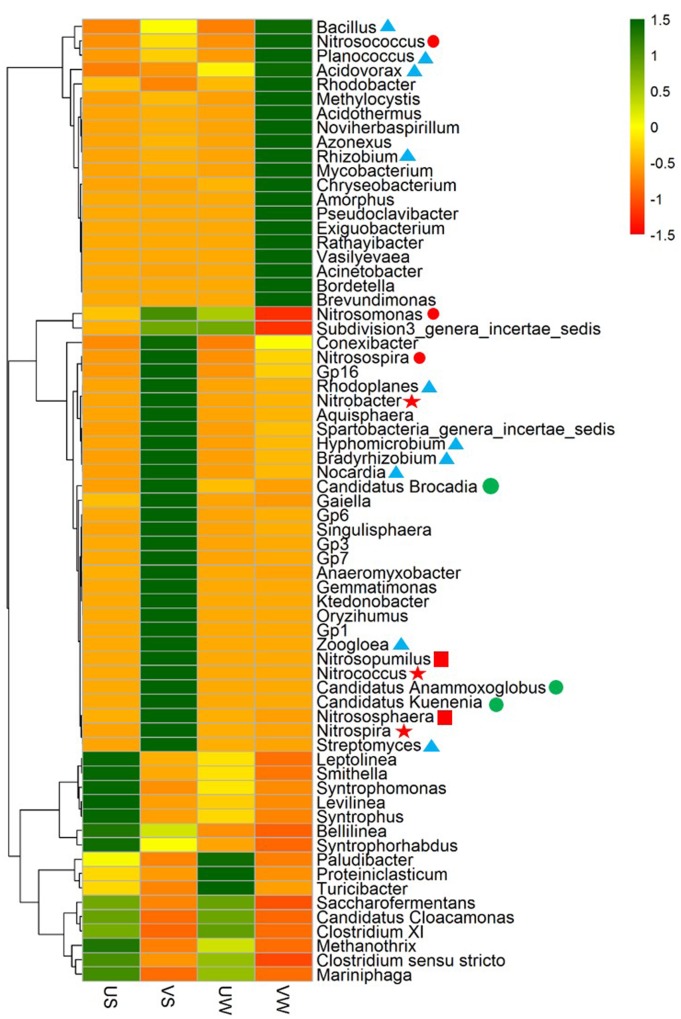
Heat map of genera in 40 samples. Colored bars indicate the range of the percentage of a genus in a sample, based on the color key at the top right. AOB: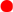; AOA: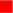; NOB: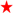; Denitrifier: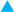; Anammox bacteria: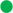. US, unvegetated sediment; VS, vegetated sediment; UW, unvegetated water; and VW, vegetated water.

To further validate the functional populations involved in N removal that are associated with *M. aquaticum*, a heat map of major genera was generated, as illustrated in **Figure 3**. For nitrifiers, the AOB *Nitrosospira* was enriched significantly by *M. aquaticum* in the sediment (*p* < 0.001). Two AOB *Nitrosomonas* and *Nitrosococcus* were also enriched. AOB in the vegetated sediment was dominated by *Nitrosospira*. The abundance of the AOA *Nitrososphaera* was significantly higher in the *M. aquaticum*-containing sediments (*p* < 0.001). *Nitrososphaera* was dominant among ammonia oxidizers. Two NOB *Nitrospira* and *Nitrobacter* were not detected in unvegetated sediment, but were significantly increased in the vegetated sediment (*p* < 0.001). Moreover, *M. aquaticum* significantly increased the abundances of the anammox bacteria *Candidatus Brocadia* and *Candidatus Kuenenia* (*p* < 0.001), while these genus were not detected in the unvegetated sediment. For the denitrifying groups, abundant denitrifiers, such as *Bradyrhizobium*, *Rhodoplanes*, *Bacillus*, *Zoogloea*, *Rhizobium*, *Acidovorax*, and *Hyphomicrobium*, were enriched significantly in the vegetated sediment (*p* < 0.001). *Bradyrhizobium* and *Rhodoplanes* were dominant among the denitrifiers. In the water containing *M. aquaticum*, among nitrifiers, the abundances of *Nitrosococcus* and *Nitrosospira* increased, whereas the abundances of *Nitrosomonas* and *Nitrososphaera* decreased. In addition, sequences were not obtained for NOB and anammox bacteria in the water with and without *M. aquaticum*. For the denitrifying group, a few denitrifiers such as *Rhizobium*, *Rhodoplanes*, and *Rhodobacter* were enriched significantly by *M. aquaticum* (*p* < 0.001).

### Change of the Sediment Physicochemical Properties and Microbial Communities Caused by *M. aquaticum*

**FIGURE 4:**
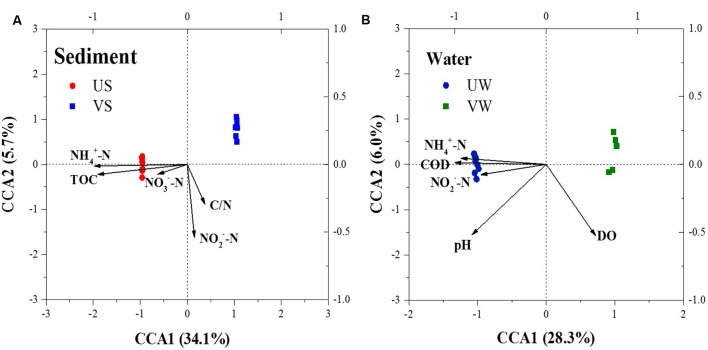
Canonical correspondence analysis (CCA) ordination plots for the first two dimensions showing the relationship between the bacterial communities (symbols) and environmental factors (arrows). **(A)** sediment; **(B)** water. US, unvegetated sediment; VS, vegetated sediment; UW, unvegetated water; and VW, vegetated water.

Compared with the unvegetated sediments, ${NH}_{4}^{+}$-N, pH, and total organic carbon (TOC) decreased significantly (*p* < 0.001), whereas ${NO}_{2}^{-}$-N increased, in the sediments containing *M. aquaticum* (**Table 1**). CCA was used to examine the relationship between the environmental variables and the microbial community composition. The results of the CCA, a significant model at the confidence level of *p* ≤ 0.001, indicated that sediment ${NH}_{4}^{+}$-N and TOC were important environmental factors controlling the microbial community structure (**Figure 4**). The importance of these environmental attributes was verified by Mantel tests based on their significant correlation (*p* ≤ 0.001) as well (**Table 2**).

**Table 1:** Chemical characteristics in the unvegetated and vegetated sediments.

Sediment	pH	${NH}_{4}^{+}$-N (g/kg)	${NO}_{2}^{-}$-N (mg/kg)	${NO}_{3}^{-}$-N (mg/kg)	TOC (g/kg)	C/N
Unvegetated	7.18 ± 0.08	0.86 ± 0.07	0.86 ± 0.32	15.35 ± 3.13	16.01 ± 0.18	8.72 ± 0.04
Vegetated	6.16 ± 0.29	0.13 ± 0.06	1.66 ± 0.35	6.45 ± 1.50	9.34 ± 0.28	10.03 ± 0.24


**Table 2:** Relationships of the microbial community structure with environmental factors as revealed by Mantel tests.

Sediment			Water		
	
Environmental attribute	*R*-value	*P*-value	Environmental attribute	*R*-value	*P-*value
pH	0.958	**0.001**	pH	0.601	**0.001**
${NH}_{4}^{+}$-N (g/kg)	0.955	**0.001**	${NH}_{4}^{+}$-N (mg/L)	0.876	**0.001**
${NO}_{2}^{-}$-N (mg/kg)	0.120	0.075	${NO}_{2}^{-}$-N (mg/L)	0.225	0.014
${NO}_{3}^{-}$-N (mg/kg)	0.046	0.179	${NO}_{3}^{-}$-N (mg/L)	0.783	**0.001**
TOC (g/kg)	0.891	**0.001**	COD (mg/L)	0.767	**0.001**
C/N	0.098	0.046	DO (mg/L)	0.263	0.007


*Significant differences (*p* ≤ 0.001) are indicated in bold. DO, dissolved oxygen.*

### Change of Microbial Interactions by *M. aquaticum*

**FIGURE 5:**
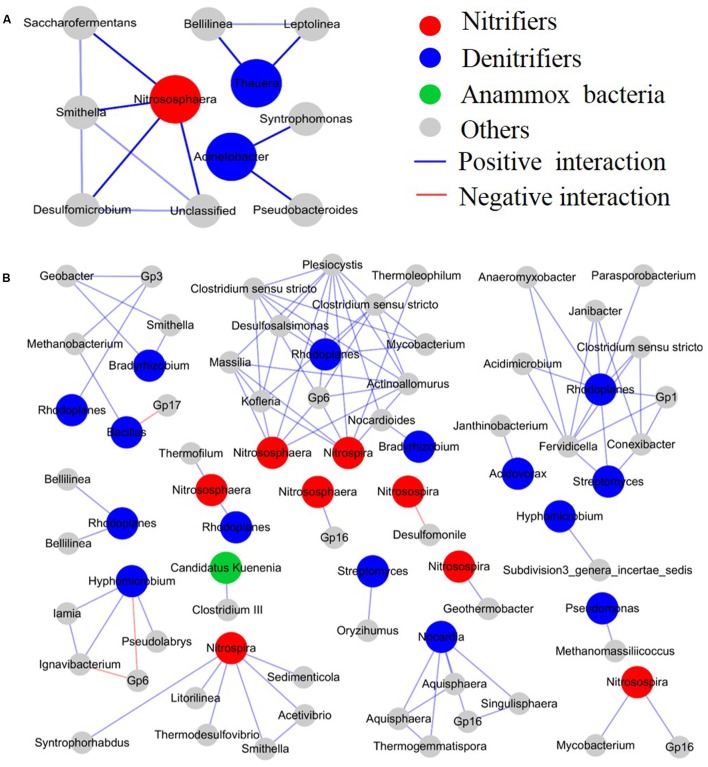
Effect of *M. aquaticum* on the microbial interactions. **(A)** Unvegetated sediment and **(B)** vegetated sediment.

Interactions among different microbial species in the CWs were calculated using a relative abundance matrix of genus-level OTUs with 97% similarity, and they were constructed in a network to describe the effect of *M. aquaticum* on the microbial interactions (**Figure 5** and Supplementary Figure 6). The structure of the identified networks with and without *M. aquaticum* differed substantially in terms of their network composition, interaction patterns (positive, zero, or negative), and node overlap, which demonstrated that *M. aquaticum* greatly altered the interactions among different microbial species. A small and simple connected module of nodes corresponded to N-cycling members in the unvegetated sediments, primarily including ammonia-oxidizing *Nitrososphaera* and denitrifiers such as *Acinetobacter* and *Thauera*, and these species did not show any interaction. However, in the sediment containing *M. aquaticum*, a large and densely connected module of nodes corresponded to members involved in N removal, primarily including ammonia-oxidizing *Nitrososphaera*, nitrite-oxidizing *Nitrospira*, anaerobic ammonia-oxidizing *Candidatus Kuenenia* and denitrifiers such as *Rhodoplanes*, *Bradyrhizobium*, and *Hyphomicrobium*. These groups were distributed widely in the vegetated sediments, and they showed positive ecological associations with different species. The positive interactions between the AOA *Nitrososphaera* and denitrifiers were stimulated by *M. aquaticum. Nitrososphaera* showed positive interactions with the anaerobic denitrifier *Rhodoplanes*. Heterotrophic denitrifiers, such as *Rhodoplanes*, *Bradyrhizobium*, and *Hyphomicrobium*, were enriched and distributed widely in the sediments containing *M. aquaticum*. Moreover, microorganisms carry out fermentation including *Clostridium* and *Smithella* were also enriched in these sediments (Ezeji et al., 2007; Wawrik et al., 2016). The enriched denitrifiers showed positive relationships with fermentative bacteria. For example, *Rhodoplanes* was frequently found to have positive interactions with the anaerobic fermentative bacteria *Clostridium*. Moreover, positive relationships among the denitrifiers were observed. However, in the vegetated water (Supplementary Figure 6), the most abundant genus, *Rhizobium*, displayed co-occurrence through positive correlations with some denitrifiers or potential denitrifiers, including *Rhodoplanes*, *Bradyrhizobium*, and *Rhodobacter*.

## Discussion

In China, discharges of COD and ammonia from livestock farms accounted for 41.9 and 41.5%, respectively, of total emission loads (Zhou et al., 2013). Our CW planted with *M. aquaticum* was used successfully for swine wastewater treatment, and it exhibited promising performance. The COD removal efficiency of 88.2% was higher than those of free water surface CWs (FWS CWs), which are commonly used to treat domestic wastewater and generally exhibit typical removal efficiencies of 70% (Zhang et al., 2014). The ${NH}_{4}^{+}$-N removal efficiency of 98.3% was higher than the reported treatment efficiencies of 40–50% in FWS CWs (Vymazal, 2007). The TN removal efficiency in our CW was 95.8%, which was higher than the TN removal efficiencies reported by other studies, such as 25% in a CW planted with *Phragmites communis* and *Scirpus validus* (Ibekwe et al., 2003), and 39.6% for 268 subsurface horizontal flow CWs in Europe (Ye and Li, 2009).

In our CW, which achieved high and stable N treatment performance during long-term operation, we used molecular biology tools to explore the mechanisms underlying its good performance. Our results revealed a remarkable enrichment of functional groups involved in N removal in sediment compared with those in the water, indicating that sediment may be of greater importance than the water as a site for N removal in the CW planted with *M. aquaticum. M. aquaticum* may affect microbial community by decreasing physicochemical factors such as ${NH}_{4}^{+}$-N and TOC. These modifications, in turn, may change the abundances and structure of nitrifying and denitrifying communities in sediment (Vila-Costa et al., 2016). In many previous studies, the ${NH}_{4}^{+}$-N concentration was always proven to be a prime factor in the N cycle in CWs (Sims et al., 2013). In the present study, the AOB *Nitrosospira* and AOA *Nitrososphaera* were significantly enriched in the vegetated sediments (*p* < 0.001), and ${NH}_{4}^{+}$-N was significantly lower in the vegetated sediments than those of the unvegetated sediments (*p* < 0.001). As previously reported, the AOB *Nitrosospira* and AOA exhibit a higher ammonia affinity and adaptability to low ammonia concentrations (Schramm et al., 1998; Herrmann et al., 2009). Thus, the low concentrations of ${NH}_{4}^{+}$-N in the vegetated sediments may favor the growth of the AOB *Nitrosospira* and the AOA *Nitrososphaera*. TOC has been considered as a favorable factor in shaping the soil microbial community composition (Shen et al., 2015). The transformation of TOC can supply labile nutrients and energy which could stimulate the growth of denitrifiers (Aldén et al., 2001).

Nitrifiers, including AOA, AOB, and NOB, were enriched significantly in the sediment containing *M. aquaticum*. The enrichment of nitrifiers may be due to the oxidized zone created by oxygen released from the roots of *Myriophyllum* spp. (Laskov et al., 2006). The qPCR results indicated *M. aquaticum* significantly increased the abundances of the archaeal and bacterial *amoA* genes. Previous studies demonstrated plant-specific effects on the abundances of archaeal and bacterial *amoA* genes. Archaeal *amoA* abundances, but not bacterial *amoA* abundances, were significantly higher in sediments containing *L. uniflora*, *Lolium perenne*, and *Festuca rubra*. While *Ceratophyllum demersum* and *Vallisneria spinulosa* increased bacterial *amoA* abundances, but not archaeal *amoA*, compared with unvegetated sediments (Herrmann et al., 2008; Zhao et al., 2014; Thion et al., 2016). In the present study, *M. aquaticum* significantly increased both archaeal and bacterial *amoA* abundances, suggesting that it maybe advantageous for both AOA and AOB. Despite our observing significant increases in both AOA and AOB, the AOA dominated numerically in all sediments containing *M. aquaticum*. This trend suggests that AOA might be better adapted to the microaerophilic conditions in the sediment, or they may benefit from the organic compounds released by roots for mixotrophic growth (Leininger et al., 2006). Other factors known to affect AOA and AOB are pH and the ammonia concentration. The greater enrichment of AOA, compared with that of AOB, may result from the significant decrease of the pH and the ammonium in the sediment containing *M. aquaticum*, as AOA are generally predominant in acidic soils and at low ammonia concentrations (Herrmann et al., 2009). The high-throughput sequencing results further indicate that the AOA that are most enriched by *M. aquaticum* was *Nitrososphaera*, and *Nitrososphaera* dominated among ammonia oxidizers. This type of archaea is found frequently in soil and the rhizosphere layer because it exhibits both facultative aerobic and anaerobic characteristics (Bouali et al., 2012). Moreover, organic carbon compounds, such as pyruvate, a constituent of plant root exudates, is essential for the high growth yields of *Nitrososphaera* and could contribute to the high proportion of *Nitrososphaera* (Tourna et al., 2011). Additionally, the enriched anammox bacteria may be due to the oxic (photosynthesis)–anoxic (submerged) interface provided by *M. aquaticum* where anammox mainly occurs (Zhu et al., 2015). Therefore, the more probable pathway for ${NH}_{4}^{+}$-N removal was through a combination of nitrifying and anammox bacteria, which enhanced the ammonium removal.

The abundance of denitrifying genes *nirK*, *nirS*, and *nosZ* was significantly higher in the sediment containing *M. aquaticum*. Thus, vegetation is an important factor controlling the denitrifier community size in the CW. Previous studies also demonstrated plant-specific effects on the abundances of the *nirK* and *nirS* genes. Of the two well-studied macrophytes, *Phragmites australis* yielded significant increases in *nirK* and *nosZ* abundances, but not *nirS* abundances, while *Typha* did not have any obvious effect on these genes in a FWS CW (García-Lledó et al., 2011). In our study, the abundance of the *nirS* gene was greater increased than that of the *nirK* gene, and the *nirS* gene outnumbered *nirK* gene in the sediments. These results indicate that there was stronger plant species effect on the *nirS* communities than on the *nirK* communities. The *nosZ* abundance did not differ significantly from the *nirK*/*nirS* abundance. As our study focus on the effect of the macrophytes on the microbial communities responsible for N removal, thus, we adopted the commonly used primers in many previous studies detecting the *nosZ* gene, and did not target representatives from clade II, which might have underestimated the actual *nosZ* gene abundance in CWs. This contrasts with previous studies demonstrating that the *nosZ* abundance is frequently lower than that of *nir* genes in the environment, as many bacterial isolates that harbor either *nirS* or *nirK* genes lack the *nosZ* gene (Henry et al., 2006; Hallin et al., 2009). The high abundance of the *nosZ* gene in our study during long-term operation of the CW may enhance the last step in the denitrification pathway, leading to a potential reduction in the emission of the greenhouse gas N_2_O (Zhi et al., 2015). The high-throughput sequencing results showed that abundant denitrifiers and potential denitrifiers were enriched significantly by *M. aquaticum*. The enriched *Acidovorax*, *Bacillus*, *Bradyrhizobium*, *Hyphomicrobium*, *Rhizobium*, and *Rhodoplanes* play important roles in wastewater treatment (Guo et al., 2013). These most increased denitrifiers belong to the Alpha-*Proteobacteria*. Within the phylum *Proteobacteria*, nearly half of all microorganisms have a complete denitrification pathway including *nir*, *nor*, and *nosZ* genes (Graf et al., 2014), which may explain the high abundances of both the *nir* and *nosZ* genes in our study. Previously, the effects of different macrophytes on the structure of the denitrifying community in the rhizosphere have been reported in freshwater wetlands (Angeloni et al., 2006; Ruiz-Rueda et al., 2009). The effect on the compositions of the denitrifying populations could be due to differences in root exudates, which have been shown to influence the abundances and diversity of microorganisms (Orwin et al., 2006). Lu et al. (2014) found that the fatty acid methyl esters and fatty acid amides from the root exudates of aquatic duckweed stimulated removal of denitrifying bacteria. Nevertheless, Henry et al. (2008) did not find strong effect on the structure and density of the denitrifying communities when using different artificial root exudates.

To elucidate the effect of *M. aquaticum* on the microbial interactions among various populations, we used RMT-based network approaches to examine the interactions of the microbial communities in response to this species. Previously, correlation networks have been successfully applied to identify bacterial interactions in marine, soil, and activated sludge samples (Ruan et al., 2006; Zhou et al., 2010; Ju et al., 2014). Our results indicated that *M. aquaticum* dramatically shifted the interactions of microbial populations. Particularly, it greatly enhanced positive ecological associations. A positive relationship is most likely due to mutualism or commensalism (Faust and Raes, 2012). The AOA *Nitrososphaera* showed a positive interaction with the denitrifier *Rhodoplanes*, suggesting that *M. aquaticum* may provide an oxygenated zone for nitrifiers growing in the sediment and enhance nitrification via oxygen release from roots, with nitrite and nitrate formed serving as substrates for denitrifiers in the adjacent anaerobic zone (Reddy et al., 1989; Ruiz-Rueda et al., 2009). The stimulated positive interactions between fermentative bacteria and the denitrifying bacteria *Rhodoplanes* suggested that the major products of anaerobic fermentation (acetate, propionate, and butyrate) may be used as carbon sources by *Rhodoplanes* (Paul et al., 1989). The enriched fermentative and denitrifying bacteria suggest that there are more available carbon sources in the vegetated sediment, which may be related to the organic carbon provided by *M. aquaticum*. The positive associations among denitrifiers such as *Rhodoplanes* and *Streptomyces* demonstrate that closely functional species have a related ecologically preference, which may promote the N removal via denitrification (Peng et al., 2014).

In this study, the effect of macrophytes on nitrifying and denitrifying bacterial communities was characterized in a pilot-scale, surface flow CW to treat high-strength swine wastewater. The CW planted with *M. aquaticum* exhibited extraordinary performance for long-term N removal without the need for costly aeration. 16S rRNA, nitrifying and denitrifying genes were enriched significantly in the sediment containing *M. aquaticum. M. aquaticum* markedly changed the structure of the microbial community, and the nitrifiers, anammox bacteria, and denitrifiers were significantly enriched in the sediment. A CCA and Mantle tests indicated that *M. aquaticum* may shift the sediment microbial community by changing the sediment chemical properties, as the ${NH}_{4}^{+}$-N and TOC concentrations were significantly lower than those of the unvegetated sediment. Positive relationships among nitrifiers, denitrifiers, and other bacteria were stimulated by *M. aquaticum* in the sediment.

## Author Contributions

HS performed the experiment, analyzed the data, and wrote the paper. FL designed the experiment, performed the experiment, and revised the paper. SX analyzed the data and revised the paper. SW analyzed the data and revised the paper. GZ revised the paper. YD analyzed the data and revised the paper. JW conceived and designed the experiment and gave comments on the paper. XZ instructed the experiment and revised the paper.

## Conflict of Interest Statement

The authors declare that the research was conducted in the absence of any commercial or financial relationships that could be construed as a potential conflict of interest.

## Footnotes

1 http://ieg2.ou.edu/MENA/

## References

[B1] Aldén L. Demoling F. Bååth E. (2001). Rapid method of determining factors limiting bacterial growth in soil. *Appl. Environ. Microbiol.* 67 1830–1838. 10.1128/AEM.67.4.1830-1838.2001 11282640 PMC92804

[B2] Angeloni N. L. Jankowski K. J. Tuchman N. C. Kelly J. J. (2006). Effects of an invasive cattail species (*Typha × glauca*) on sediment nitrogen and microbial community composition in a freshwater wetland. *FEMS Microbiol. Lett.* 263 86–92. 10.1111/j.1574-6968.2006.00409.x 16958855

[B3] Beman J. M. Arrigo K. R. Matson P. A. (2005). Agricultural runoff fuels large phytoplankton blooms in vulnerable areas of the ocean. *Nature* 434 211–214. 10.1038/nature03370 15758999

[B4] Bodelier P. Libochant J. A. Blom C. Laanbroek H. J. (1996). Dynamics of nitrification and denitrification in root-oxygenated sediments and adaptation of ammonia-oxidizing bacteria to low-oxygen or anoxic habitats. *Appl. Environ. Microbiol.* 62 4100–4107. 16535441 10.1128/aem.62.11.4100-4107.1996 PMC1388979

[B5] Bouali M. Zrafi-Nouira I. Bakhrouf A. Le Paslier D. Chaussonnerie S. Ammar E. (2012). The structure and spatio-temporal distribution of the Archaea in a horizontal subsurface flow constructed wetland. *Sci. Total Environ.* 435 465–471. 10.1016/j.scitotenv.2012.07.047 22885352

[B6] Caporaso J. G. Lauber C. L. Walters W. A. Berg-Lyons D. Huntley J. Fierer N. (2012). Ultra-high-throughput microbial community analysis on the Illumina HiSeq and MiSeq platforms. *ISME J.* 6 1621–1624. 10.1038/ismej.2012.8 22402401 PMC3400413

[B7] Cronk J. K. (1996). Constructed wetlands to treat wastewater from dairy and swine operations: a review. *Agric. Ecosyst. Environ.* 58 97–114. 10.1016/0167-8809(96)01024-9

[B8] Deng Y. Jiang Y.-H. Yang Y. He Z. Luo F. Zhou J. (2012). Molecular ecological network analyses. *BMC Bioinformat.* 13:113 10.1186/1471-2105-13-113 PMC3428680 22646978

[B9] Dong X. Reddy G. B. (2010). Soil bacterial communities in constructed wetlands treated with swine wastewater using PCR-DGGE technique. *Bioresour. Technol.* 101 1175–1182. 10.1016/j.biortech.2009.09.071 19822421

[B10] Edgar R. C. (2013). UPARSE: highly accurate OTU sequences from microbial amplicon reads. *Nat. Methods* 10 996–998. 10.1038/nmeth.2604 23955772

[B11] Ezeji T. Qureshi N. Blaschek H. P. (2007). Butanol production from agricultural residues: impact of degradation products on *Clostridium beijerinckii* growth and butanol fermentation. *Biotechnol. Bioeng.* 97 1460–1469. 10.1002/bit.21373 17274071

[B12] Faust K. Raes J. (2012). Microbial interactions: from networks to models. *Nat. Rev. Microbiol.* 10 538–550. 10.1038/nrmicro2832 22796884

[B13] Federation W. E. Association A. P. H. (2005). *Standard Methods for the Examination of Water and Wastewater.* Washington, DC: American Public Health Association.

[B14] García-Lledó A. Vilar-Sanz A. Trias R. Hallin S. Bañeras L. (2011). Genetic potential for N2O emissions from the sediment of a free water surface constructed wetland. *Water Res.* 45 5621–5632. 10.1016/j.watres.2011.08.025 21920580

[B15] Graf D. R. Jones C. M. Hallin S. (2014). Intergenomic comparisons highlight modularity of the denitrification pathway and underpin the importance of community structure for N2O emissions. *PLOS ONE* 9:e114118 10.1371/journal.pone.0114118 PMC4250227 25436772

[B16] Guo F. Ju F. Cai L. Zhang T. (2013). Taxonomic precision of different hypervariable regions of 16S rRNA gene and annotation methods for functional bacterial groups in biological wastewater treatment. *PLOS ONE* 8:e76185 10.1371/journal.pone.0076185 PMC3797802 24146837

[B17] Hallin S. Jones C. M. Schloter M. Philippot L. (2009). Relationship between N-cycling communities and ecosystem functioning in a 50-year-old fertilization experiment. *ISME J.* 3 597–605. 10.1038/ismej.2008.128 19148144

[B18] He T. Guan W. Luan Z. Xie S. (2016). Spatiotemporal variation of bacterial and archaeal communities in a pilot-scale constructed wetland for surface water treatment. *Appl. Microbiol. Biotechnol.* 100 1479–1488. 10.1007/s00253-015-7072-5 26496919

[B19] Henry S. Bru D. Stres B. Hallet S. Philippot L. (2006). Quantitative detection of the nosZ gene, encoding nitrous oxide reductase, and comparison of the abundances of 16S rRNA, narG, nirK, and nosZ genes in soils. *Appl. Environ. Microbiol.* 72 5181–5189. 10.1128/AEM.00231-06 16885263 PMC1538733

[B20] Henry S. Texier S. Hallet S. Bru D. Dambreville C. Chèneby D. (2008). Disentangling the rhizosphere effect on nitrate reducers and denitrifiers: insight into the role of root exudates. *Environ. Microbiol.* 10 3082–3092. 10.1111/j.1462-2920.2008.01599.x 18393993

[B21] Herrmann M. Saunders A. M. Schramm A. (2008). Archaea dominate the ammonia-oxidizing community in the rhizosphere of the freshwater macrophyte Littorella uniflora. *Appl. Environ. Microbiol.* 74 3279–3283. 10.1128/AEM.02802-07 18344332 PMC2394948

[B22] Herrmann M. Saunders A. M. Schramm A. (2009). Effect of lake trophic status and rooted macrophytes on community composition and abundance of ammonia-oxidizing prokaryotes in freshwater sediments. *Appl. Environ. Microbiol.* 75 3127–3136. 10.1128/AEM.02806-08 19304820 PMC2681662

[B23] Ibekwe A. M. Grieve C. M. Lyon S. R. (2003). Characterization of microbial communities and composition in constructed dairy wetland wastewater effluent. *Appl. Environ. Microbiol.* 69 5060–5069. 10.1128/AEM.69.9.5060-5069.2003 12957887 PMC194942

[B24] Ju F. Xia Y. Guo F. Wang Z. Zhang T. (2014). Taxonomic relatedness shapes bacterial assembly in activated sludge of globally distributed wastewater treatment plants. *Environ. Microbiol.* 16 2421–2432. 10.1111/1462-2920.12355 24329969

[B25] Karjalainen H. Stefansdottir G. Tuominen L. Kairesalo T. (2001). Do submersed plants enhance microbial activity in sediment? *Aquat. Bot.* 69 1–13. 10.1016/S0304-3770(00)00138-8

[B26] Kong Y. (2011). Btrim: a fast, lightweight adapter and quality trimming program for next-generation sequencing technologies. *Genomics* 98 152–153. 10.1016/j.ygeno.2011.05.009 21651976

[B27] Laskov C. Horn O. Hupfer M. (2006). Environmental factors regulating the radial oxygen loss from roots of *Myriophyllum spicatum* and *Potamogeton crispus*. *Aquat. Bot.* 84 333–340. 10.1016/j.aquabot.2005.12.005

[B28] Legendre P. Legendre L. F. (2012). *Numerical Ecology.* Amsterdam: Elsevier.

[B29] Leininger S. Urich T. Schloter M. Schwark L. Qi J. Nicol G. (2006). Archaea predominate among ammonia-oxidizing prokaryotes in soils. *Nature* 442 806–809. 10.1038/nature04983 16915287

[B30] Liu F. Zhang S. Wang Y. Li Y. Xiao R. Li H. (2016). Nitrogen removal and mass balance in newly-formed *Myriophyllum aquaticum* mesocosm during a single 28-day incubation with swine wastewater treatment. *J. Environ. Manage.* 166 596–604. 10.1016/j.jenvman.2015.11.020 26607567

[B31] Lu Y. Zhou Y. Nakai S. Hosomi M. Zhang H. Kronzucker H. J. (2014). Stimulation of nitrogen removal in the rhizosphere of aquatic duckweed by root exudate components. *Planta* 239 591–603. 10.1007/s00425-013-1998-6 24271005 PMC3928532

[B32] Magoč T. Salzberg S. L. (2011). FLASH: fast length adjustment of short reads to improve genome assemblies. *Bioinformatics* 27 2957–2963. 10.1093/bioinformatics/btr507 21903629 PMC3198573

[B33] Ministry of Environmental Protection National Bureau of Statistics of China Ministry of Agriculture (2010). *The Bulletin on the First National Census on Pollution Sources.* Beijing: Ministry of Environmental Protection.

[B34] Orwin K. H. Wardle D. A. Greenfield L. G. (2006). Ecological consequences of carbon substrate identity and diversity in a laboratory study. *Ecology* 87 580–593. 10.1890/05-0383 16602288

[B35] Ottosen L. D. M. Risgaard-Petersen N. Nielsen L. P. (1999). Direct and indirect measurements of nitrification and denitrification in the rhizosphere of aquatic macrophytes. *Aquat. Microb. Ecol.* 19 81–91. 10.3354/ame019081

[B36] Patra A. K. Abbadie L. Clays-Josserand A. Degrange V. Grayston S. J. Guillaumaud N. (2006). Effects of management regime and plant species on the enzyme activity and genetic structure of N-fixing, denitrifying and nitrifying bacterial communities in grassland soils. *Environ. Microbiol.* 8 1005–1016. 10.1111/j.1462-2920.2006.00992.x 16689721

[B37] Paul J. Beauchamp E. Trevors J. (1989). Acetate, propionate, butyrate, glucose, and sucrose as carbon sources for denitrifying bacteria in soil. *Can. J. Microbiol.* 35 754–759. 10.1139/m89-126

[B38] Pelissari C. Ávila C. Trein C. M. García J. de Armas R. D. Sezerino P. H. (2017). Nitrogen transforming bacteria within a full-scale partially saturated vertical subsurface flow constructed wetland treating urban wastewater. *Sci. Total Environ.* 574 390–399. 10.1016/j.scitotenv.2016.08.207 27639475

[B39] Peng X. Guo F. Ju F. Zhang T. (2014). Shifts in the microbial community, nitrifiers and denitrifiers in the biofilm in a full-scale rotating biological contactor. *Environ. Sci. Technol.* 48 8044–8052. 10.1021/es5017087 24936907

[B40] Petersen N. R. Jensen K. (1997). Nitrification and denitrification in the rhizosphere of the aquatic macrophyte *Lobelia dortmanna* L. *Limnol. Oceanogr.* 42 529–537. 10.4319/lo.1997.42.3.0529

[B41] Philippot L. Piutti S. Martin-Laurent F. Hallet S. Germon J. C. (2002). Molecular analysis of the nitrate-reducing community from unplanted and maize-planted soils. *Appl. Environ. Microbiol.* 68 6121–6128. 10.1128/AEM.68.12.6121-6128.2002 12450836 PMC134418

[B42] Philippot L. Raaijmakers J. M. Lemanceau P. Van Der Putten W. H. (2013). Going back to the roots: the microbial ecology of the rhizosphere. *Nat. Rev. Microbiol.* 11 789–799. 10.1038/nrmicro3109 24056930

[B43] Reddy K. Patrick W. Lindau C. (1989). Nitrification-denitrification at the plant root-sediment interface in wetlands. *Limnol. Oceanogr.* 34 1004–1013. 10.4319/lo.1989.34.6.1004

[B44] Ruan Q. Dutta D. Schwalbach M. S. Steele J. A. Fuhrman J. A. Sun F. (2006). Local similarity analysis reveals unique associations among marine bacterioplankton species and environmental factors. *Bioinformatics* 22 2532–2538. 10.1093/bioinformatics/btl417 16882654

[B45] Ruiz-Rueda O. Hallin S. Bañeras L. (2009). Structure and function of denitrifying and nitrifying bacterial communities in relation to the plant species in a constructed wetland. *FEMS Microbiol. Ecol.* 67 308–319. 10.1111/j.1574-6941.2008.00615.x 19049502

[B46] Schramm A. de Beer D. Wagner M. Amann R. (1998). Identification and activities in situ of nitrosospiraand *Nitrospira* spp. as dominant populations in a nitrifying fluidized bed reactor. *Appl. Environ. Microbiol.* 64 3480–3485. 9726900 10.1128/aem.64.9.3480-3485.1998 PMC106750

[B47] Shen Z. Zhou Y. Liu J. Xiao Y. Cao R. Wu F. (2015). Enhanced removal of nitrate using starch/PCL blends as solid carbon source in a constructed wetland. *Bioresour. Technol.* 175 239–244. 10.1016/j.biortech.2014.10.006 25459828

[B48] Sims A. Zhang Y. Gajaraj S. Brown P. B. Hu Z. (2013). Toward the development of microbial indicators for wetland assessment. *Water Res.* 47 1711–1725. 10.1016/j.watres.2013.01.023 23384515

[B49] Stottmeister U. Wießner A. Kuschk P. Kappelmeyer U. Kästner M. Bederski O. (2003). Effects of plants and microorganisms in constructed wetlands for wastewater treatment. *Biotechnol. Adv.* 22 93–117. 10.1016/j.biotechadv.2003.08.010 14623046

[B50] Thion C. E. Poirel J. D. Cornulier T. De Vries F. T. Bardgett R. D. Prosser J. I. (2016). Plant nitrogen-use strategy as a driver of rhizosphere archaeal and bacterial ammonia oxidiser abundance. *FEMS Microbiol. Ecol.* 92:fiw091 10.1093/femsec/fiw091 27130939

[B51] Tourna M. Stieglmeier M. Spang A. Könneke M. Schintlmeister A. Urich T. (2011). Nitrososphaera viennensis, an ammonia oxidizing archaeon from soil. *Proc. Natl. Acad. Sci. U.S.A.* 108 8420–8425. 10.1073/pnas.1013488108 21525411 PMC3100973

[B52] Vartapetian B. B. Jackson M. B. (1997). Plant adaptations to anaerobic stress. *Ann. Bot.* 79(Suppl. 1), 3–20. 10.1093/oxfordjournals.aob.a010303

[B53] Vila-Costa M. Pulido C. Chappuis E. Calviño A. Casamayor E. O. Gacia E. (2016). Macrophyte landscape modulates lake ecosystem-level nitrogen losses through tightly coupled plant-microbe interactions. *Limnol. Oceanogr.* 61 78–88. 10.1002/lno.10209

[B54] Vymazal J. (2007). Removal of nutrients in various types of constructed wetlands. *Sci. Total Environ.* 380 48–65. 10.1016/j.scitotenv.2006.09.014 17078997

[B55] Vymazal J. (2011). Constructed Wetlands for wastewater treatment: five decades of experience†. *Environ. Sci. Technol.* 45 61–69. 10.1021/es101403q 20795704

[B56] Wang Q. Garrity G. M. Tiedje J. M. Cole J. R. (2007). Naive Bayesian classifier for rapid assignment of rRNA sequences into the new bacterial taxonomy. *Appl. Environ. Microbiol.* 73 5261–5267. 10.1128/AEM.00062-07 17586664 PMC1950982

[B57] Wawrik B. Marks C. R. Davidova I. A. McInerney M. J. Pruitt S. Duncan K. E. (2016). Methanogenic paraffin degradation proceeds via alkane addition to fumarate by ‘*Smithella*’spp. mediated by a syntrophic coupling with hydrogenotrophic methanogens. *Environ. Microbiol.* 18 2604–2619. 10.1111/1462-2920.13374 27198766

[B58] Wu Y. Rui H. Yang X. Fang X. Xi C. Di Y. (2016). Correlating microbial community with physicochemical indices and structures of a full-scale integrated constructed wetland system. *Appl. Microbiol. Biotechnol.* 100 6917–6926. 10.1007/s00253-016-7526-4 27100531

[B59] Yang Y. Gao Y. Wang S. Xu D. Yu H. Wu L. (2014). The microbial gene diversity along an elevation gradient of the Tibetan grassland. *ISME J.* 8 430–440. 10.1038/ismej.2013.146 23985745 PMC3906809

[B60] Ye F. Li Y. (2009). Enhancement of nitrogen removal in towery hybrid constructed wetland to treat domestic wastewater for small rural communities. *Ecol. Engin.* 35 1043–1050. 10.1016/j.ecoleng.2009.03.009

[B61] Zhang D. Q. Jinadasa K. Gersberg R. M. Liu Y. Ng W. J. Tan S. K. (2014). Application of constructed wetlands for wastewater treatment in developing countries–a review of recent developments (2000–2013). *J. Environ. Manage.* 141 116–131. 10.1016/j.jenvman.2014.03.015 24784754

[B62] Zhao D.-Y. Luo J. Zeng J. Wang M. Yan W.-M. Huang R. (2014). Effects of submerged macrophytes on the abundance and community composition of ammonia-oxidizing prokaryotes in a eutrophic lake. *Environ. Sci. Pollut. Res.* 21 389–398. 10.1007/s11356-013-1909-1 23784056

[B63] Zhi W. Yuan L. Ji G. He C. (2015). Enhanced long-term nitrogen removal and its quantitative molecular mechanism in tidal flow constructed wetlands. *Environ. Sci. Technol.* 49 4575–4583. 10.1021/acs.est.5b00017 25781063

[B64] Zhou J. Deng Y. Luo F. He Z. Tu Q. Zhi X. (2010). Functional molecular ecological networks. *mBio* 1:e00169–10 10.1128/mBio.00169-10 PMC2953006 20941329

[B65] Zhou J. Deng Y. Luo F. He Z. Yang Y. (2011). Phylogenetic molecular ecological network of soil microbial communities in response to elevated CO2. *mBio* 2:e00122–11 10.1128/mBio.00122-11 PMC3143843 21791581

[B66] Zhou L.-J. Ying G.-G. Liu S. Zhang R.-Q. Lai H.-J. Chen Z.-F. (2013). Excretion masses and environmental occurrence of antibiotics in typical swine and dairy cattle farms in China. *Sci. Total Environ.* 444 183–195. 10.1016/j.scitotenv.2012.11.087 23268145

[B67] Zhu G. Wang S. Zhou L. Wang Y. Zhao S. Xia C. (2015). Ubiquitous anaerobic ammonium oxidation in inland waters of China: an overlooked nitrous oxide mitigation process. *Sci. Rep.* 5:17306 10.1038/srep17306 PMC4661425 26610807

