# Supplementary material for: Myriophyllum aquaticum Constructed Wetland Effectively Removes Nitrogen in Swine Wastewater
Source: Front Microbiol. 2017 Oct 6;8:1932. doi: 10.3389/fmicb.2017.01932 (PMC5635519; doi:10.3389/fmicb.2017.01932)
Supplement: Supplementary file 1 [file Data_Sheet_1.docx]

Supplementary Material

***Myriophyllum aquaticum* constructed wetland effectively removes nitrogen in swine wastewater**

Haishu Sun, ^1,3*^ Feng Liu, ^2*^ Shengjun Xu, ^1,3^ Shanghua Wu, ^1,3^ Guoqiang Zhuang,^1,3^

Ye Deng, ^1,3^ Jinshui Wu,^2**^ and Xuliang Zhuang ^1,3**^

^1^Key Laboratory of Environmental Biotechnology, Research Center for Eco-Environmental Sciences, Chinese Academy of Sciences, Beijing 100085, China

^2^Key Laboratory of Agro-ecological Processes in Subtropical Regions, Institute of Subtropical Agriculture, Chinese Academy of Sciences, Hunan 410125, China

^3^College of Resources and Environment, University of Chinese Academy of sciences, Beijing 100049, China

*H.S.S and F.L contributed equally to this paper.

**Corresponding author: [jswu@isa.ac.cn](mailto:jswu@isa.ac.cn) (JS Wu) and [xlzhuang@rcees.ac.cn](mailto:xlzhuang@rcees.ac.cn) (XL Zhuang)

# Supplementary Figures and Tables

## Supplementary Figures


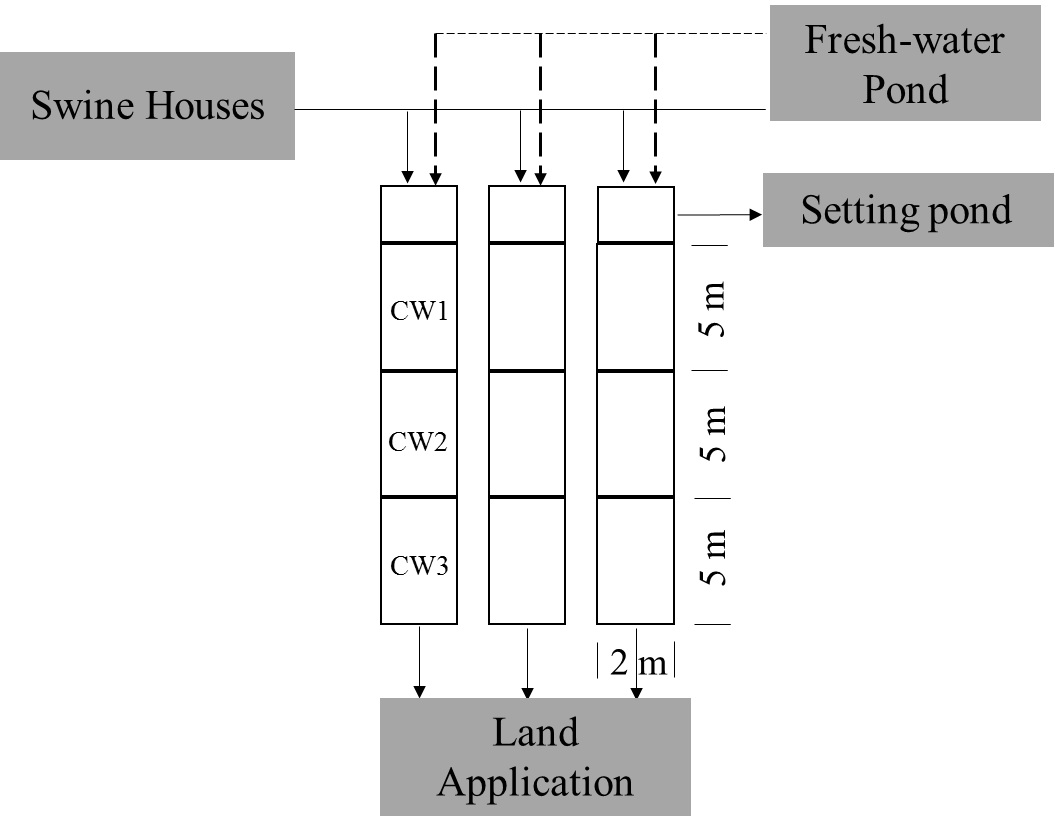


**Supplementary Figure 1.** Schematic diagram of constructed wetlands for treating swine wastewater.

**Supplementary Figure 2.** The abundances of the bacterial 16S *rRNA*, archaeal and bacterial *amoA*, *nirK*, *nirS*, and *nosZ*. UW: water without *M.aquaticum* planting; VFW: water with *M.aquaticum* in the first-stage wetland; VSW: water with *M.aquaticum* in the second-stage wetland; VTW: water with *M.aquaticum* in the third-stage wetland.

**Supplementary Figure 3.** Principal coordinate analysis (PCoA) of the unweighted UniFrac distances between all samples.

**Supplementary Figure 4.** Taxonomic distributions of bacterial phyla in the sediment and water. Each bar represent the average value of ten replicates in each treatment. US, unvegetated sediment, VS, vegetated sediment, UW, unvegetated water, VW, vegetated water.

 **Supplementary Figure 5.** Taxonomic distributions of bacterial class in the sediment and water column. Each bar represent the average value of ten replicates in each treatment. US, unvegetated sediment, VS, vegetated sediment, UW, unvegetated water, VW, vegetated water.


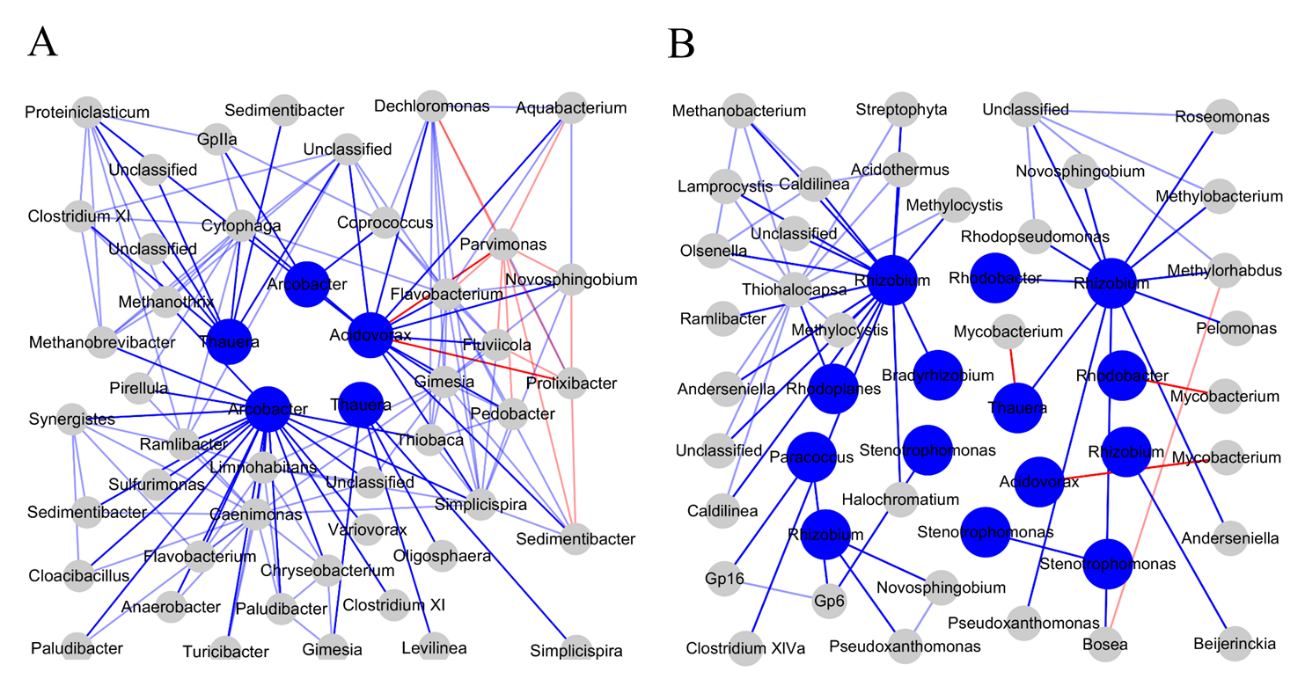


**Supplementary Figure 6.** Effect of *Myriophyllum aquaticum* on the microbial interactions. (A) unvegetated water; and (B) vegetated water.

## Supplementary Tables

**Supplementary Table 1.** Swine wastewater characteristic.

| Parameters | COD  (mg L^-1^) | NH_4_^+^-N  (mg L^-1^) | NO_3_^—^N  (mg L^-1^) | TN  (mg L^-1^) | TP  (mg L^-1^) |
| --- | --- | --- | --- | --- | --- |
|  | 500–1200 | 220–550 | 0.15–0.88 | 380–650 | 80-150 |

**Supplementary Table 2.** Primer sets and qPCR programs used for target gene amplification.

| Target gene | Primer set | Reference | Thermal cycling profile | No. of cycles |  |
| --- | --- | --- | --- | --- | --- |
| 16S rRNA | 515F  806R | ^1^ | 95℃/10s, 60℃/30s, 72℃/60s | 40 |  |
| amoA(AOA) | Arch-amoAF | ^2^ | 95℃/5s, 55℃/30s, 72℃/60s | 40 |  |
|  | Arch-amoAR |  |  |  |  |
| amoA(AOB) | amoA-1F | ^2^ | 95℃/10s, 55℃/30s, 72℃/30s | 40 |  |
|  | amoA-2R |  |  |  |  |
| nirK | F1aCu | ^3^ | 95℃/5s, 57℃/30s, 72℃/30s | 40 |  |
|  | R3Cu |  |  |  |  |
| nirS | cd3aF | ^3, 4^ | 95℃/10s, 60℃/30s, 72℃/60s | 40 |  |
|  | R3cd |  |  |  |  |
| nosZ | nosZF | ^3, 5^ | 95℃/10s, 55℃/30s, 72℃/60s | 40 |  |
|  | nosZ1622R |  |  |  | |

**Supplementary Table 3.** α-diversity for the bacterial communities in water and sediment samples.

| Treatment | Chao 1 | Observed species | PD whole tree | Shannon | Invsimpson |
| --- | --- | --- | --- | --- | --- |
| US | 3514 | 1347.9 | 76.2 | 5.27 | 43.99 |
| VS | 7606 | 2901.9 | 113.7 | 7.07 | 378.42 |
| UW | 3587 | 1374.3 | 78.6 | 5.22 | 34.36 |
| VW | 2649 | 908.2 | 49.1 | 4.29 | 21.98 |

US, unvegetated sediment, VS, vegetated sediment, UW, unvegetated water, VW, vegetated water.

**Supplementary references**

(1) Caporaso, J. G.; Lauber, C. L.; Walters, W. A.; Berg-Lyons, D.; Lozupone, C. A.; Turnbaugh, P. J.; Fierer, N.; Knight, R. Global patterns of 16S rRNA diversity at a depth of millions of sequences per sample. *Proceedings of the National Academy of Sciences*. **2011**, *108* (Supplement 1), 4516-4522.

(2) Rotthauwe, J. H.; Witzel, K. P.; Liesack, W. The Ammonia Monooxygenase Structural Gene amoA as a Functional Marker: Molecular Fine-Scale Analysis of Natural Ammonia-Oxidizing Populations. *Applied & Environmental Microbiology*. **1997**, *63* (12), 4704-12.

(3) Throbäck, I. N.; Enwall, K.; Jarvis, Å.; Hallin, S. Reassessing PCR primers targeting nirS , nirK and nosZ genes for community surveys of denitrifying bacteria with DGGE. *FEMS microbiology ecology*. **2004**, *49* (3), 401-417.

(4) Michotey, V.; Méjean, V.; Bonin, P. Comparison of methods for quantification of cytochrome cd(1)-denitrifying bacteria in environmental marine samples. *Applied & Environmental Microbiology*. **2000**, *66* (4), 1564-71.

(5) Kloos, K.; Mergel, A. C.; Bothe, H. Denitrification within the genus Azospirillum and other associativebacteria. *Functional Plant Biology*. **2001**, *28* (9), 991-998.
